# Target women: Equity in access to mHealth technology in a non-communicable disease care intervention in Kenya

**DOI:** 10.1371/journal.pone.0220834

**Published:** 2019-09-11

**Authors:** Christine Ngaruiya, Samuel Oti, Steven van de Vijver, Catherine Kyobutungi, Caroline Free

**Affiliations:** 1 Department of Emergency Medicine, Yale School of Medicine, New Haven, Connecticut, United States of America; 2 International Development Research Centre, Nairobi, Kenya; 3 Amsterdam Institute for Global Health and Development, Amsterdam, The Netherlands; 4 African Population Health Research Center, Nairobi, Kenya; 5 Department of Population Health, London School of Hygiene and Tropical Medicine, London, United Kingdom; Anglia Ruskin University, UNITED KINGDOM

## Abstract

**Background:**

Non-Communicable Diseases (NCDs) constitute 40 million deaths annually. Eighty-percent of these deaths occur in Low- and Middle-Income Countries. MHealth provides a potentially highly effective modality for global public health, however access is poorly understood. The objective of our study was to assess equity in access to mHealth in an NCD intervention in Kenya.

**Methods:**

This is a secondary analysis of a complex NCD intervention targeting slum residents in Kenya. The primary outcomes were: willingness to receive SMS, whether SMS was received, and access to SMS compared to alternative health information modalities. Age, sex, level of education, level of income, type of work, number of hours worked, and home environment were explanatory variables considered. Multivariable regression analyses were used to test for association using likelihood ratio testing.

**Results:**

7,618 individual participants were included in the analysis. The median age was 44 years old. Majority (75%, n = 3,691/ 4,927) had only attended up to primary (elementary) school. Majority reported earning “KShs 7,500 or greater” (27%, n = 1,276/ 4,736). Age and level of income had evidence of association with willingness to receive SMS, and age, sex and number of hours work with whether SMS was received. SMS was the health information modality with highest odds of being accessed in older age groups (OR 4.70, 8.72 and 28.89, for age brackets 60–69, 70–79 and 80 years or older, respectively), among women (OR = 1.86, 95% CI 1.19–2.89), and second only to *Baraazas* (community gatherings) among those with lowest income.

**Conclusion:**

Women had the greatest likelihood of receiving SMS. SMS performed equitably well amongst marginalized populations (elderly, women, and low-income) as compared to alternative health information modalities, though sensitization prior to implementation of mHealth interventions may be needed. These findings provide guidance for developing mHealth interventions targeting marginalized populations in these settings.

## Introduction

Non-Communicable Diseases (NCDs) annually constitute around 40 million deaths every year, which is more than 60% of deaths worldwide [1, 2]. Furthermore, current disease trends suggest significant worsening of the situation over the next decade, with the WHO projecting 55 million deaths from NCDs annually by 2030 if there is no urgent action taken [3]. NCDs have surpassed communicable diseases as the lead cause of death in all continents except Africa, where NCD-related deaths are nevertheless projected to surpass deaths from communicable diseases, maternal and perinatal conditions, and nutritional deficiencies by 2030 [2–7]. This “double burden of disease”, involving both communicable and non-communicable disease, adds economic stress to already strained health systems and affected individuals, and threatens advances made with communicable disease control efforts [8–10]. Immediate and evidence-based action targeting NCDs is critically needed.

In addressing NCDs, it is necessary to consider equity in access to and outcomes from interventions used. In the 2010 WHO book, “Equity, social determinants and public health programmes,” and the 1990 Whitehead book, five major categories are outlined as indicators of equity [11–12]. Firstly, “socioeconomic status” is defined as being unique to individual cultural settings and pertains to attributes like gender, education, income, and occupation. Secondly, “differential exposure”, is defined as being inherently tied to socioeconomic status, and includes factors such as housing and work environment. Thirdly, “differential vulnerability” pertains to increased affliction from health risk factors given an already disadvantaged state such as social exclusion, low income or malnutrition. Fourthly, “differential health care outcomes” refers to disparities in health that are as a result of healthcare provided by an inequitable health system. Finally, “differential consequences” applies to the downward trend in health status that occurs as a result of decreased economic productivity from health limitations [12].

MHealth provides a potentially highly effective modality for global public health given geographic reach through increasingly widespread access to mobile phones and marginal cost [13–15]. Its use is of particular interest with conditions requiring long-term management given ability to capture those lost to follow-up, send reminders, and disseminate critical health education. However, as with any intervention or new technology, equity should be kept in mind, particularly given limited resources and funding for health [16]. It is important to consider whether mHealth is accessible, and particularly for marginalized populations who are challenging to reach by existing modalities and who already have worse health statuses than their counterparts [12, 17, 18]. To our knowledge, this will be the first study done to assess equity in access to mHealth in the developing country setting.

## Materials and methods

This study is a secondary analysis of the African Population and Health Research Center (APHRC) SCALE UP complex intervention targeting slum residents with NCDs in Kenya. Our goal was to evaluate equity in access to mHealth technology (SMS clinic appointment reminders and health education messages) in this intervention using indicators of equity from the Whitehead framework described earlier [12, 19, 20]. For this analysis, we used results from cross-sectional surveys conducted during the intervention. Our objectives were to: explore equity in respondent willingness to receive mobile messages, explore equity in likelihood of respondent receipt of mobile messages, and explore equity in access to SMS compared to alternative sources of health information. All data is available through APHRC, and can be accessed at this website: http://microdataportal.aphrc.org/index.php/catalog/79.

### Original study: The SCALE UP intervention

**Fig 1:**
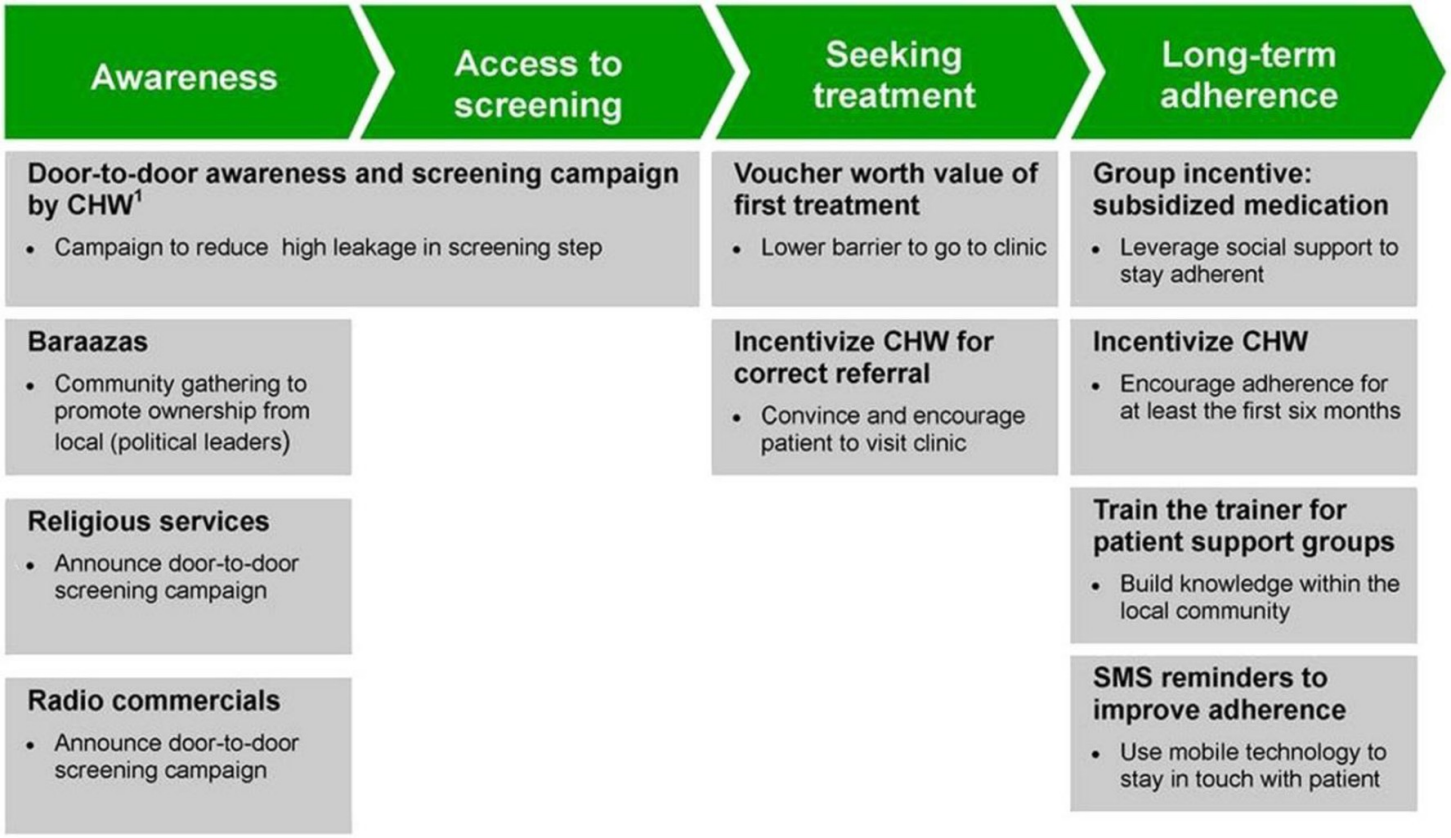
APHRC SCALE UP trial intervention components (with permission, from Oti et al 2013).

In 2012, the APHRC launched a quasi-experimental trial called SCALE UP to assess impact and cost effectiveness of a complex intervention to improve follow up for NCD care in two of the largest slums in Kenya (Korogocho and Viwandani) as comparison sites [21]. The APHRC has had different projects there since 2002 [22]. The intervention is summarized in **Fig 1**, with study recruitment and further description of SCALE UP published elsewhere [21, 23].

### Study design for secondary analysis

The data for our study originated from four cross-sectional surveys performed along each phase of the intervention. These surveys were: a *Population Baseline*, *a Clinic Baseline*, *a Clinic Endline* and a *Population Endline* survey. During the “Awareness” and screening period shown in **Fig 1**, contacts were recruited for the *Population Baseline* survey. The *Population Baseline* survey included sociodemographic factors, history of NCD diagnoses, exposure to NCD lifestyle risk factors and health-seeking behavior. The *Clinic Baseline* survey was done on those that attended the clinic as a result of a referral provided during the “Awareness Phase” of the intervention. The *Clinic Endline* survey was completed after one year, at the end of the intervention. The *Clinic* surveys assessed sociodemographic factors, NCD history and management of disease, and access to health information prior to and unique to the intervention. Finally, the *Population Endline* survey, a sub-sample of those surveyed in the *Population Baseline* survey assessed similar outcomes to the *Baseline* version.

### Equity factors affecting access to mHealth

**Fig 2:**
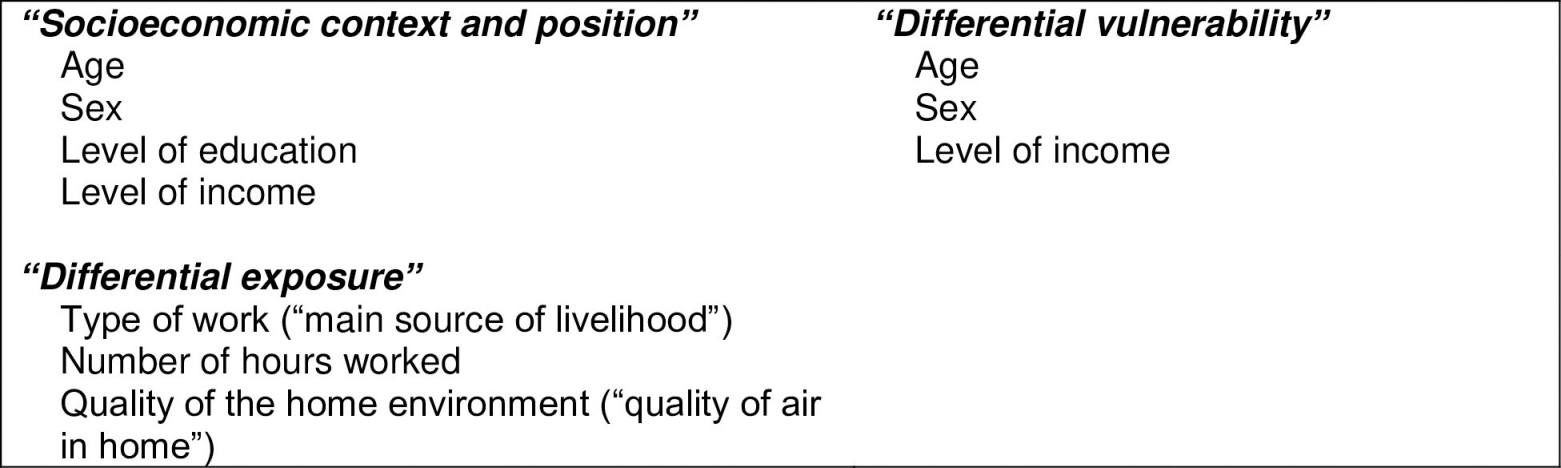
Determinants affecting access to mHealth: Indicators used from SCALE UP study surveys [11, 21].

We explored three categories of equity outlined by the WHO and Whitehead books [11, 12] previously discussed, with indicators identified from SCALE UP surveys (see **Fig 2**). These survey responses on indicators of equity were self-reported.

Firstly, “socioeconomic context and position” and “differential vulnerability” were indicated by age, sex, level of education and level of income (see **Fig 2**). The hypotheses we were interested in addressing were: that older respondents are less likely to be willing to receive SMS due to lack of familiarity/ comfort with technology; that female respondents are less likely to be willing to receive SMS due to issues such as lack of empowerment, decision-making, authority or autonomy; that those with lower levels of education are less likely to be willing to receive SMS due to lack of familiarity with technology or illiteracy [12, 24], and that those with lower levels of income are less likely to be willing to receive SMS due to concerns about economic costs related to receiving text messages or accessing care.

Secondly, “differential exposure” was assessed using indicators from the surveys on: type of work, number of hours worked, and the quality of the home environment. The hypotheses of interest with regards to exposures were: that respondents with longer work hours are less likely to be willing to or to have received SMS due to time constraints, that those with more manual types of jobs (attributed to level of busy-ness and lower SES) are less likely to be willing to or to have received SMS [25, 26], and that those with poorer indicators of the quality of their homes are less likely to have received SMS due to those with poorer indicators of social environment having less equity or access to resources [27].

Finally, we assessed equity in accessing SMS as compared to other aspects of the intervention and alternative outside sources of health information by assessing the outcome of modality of health information reported. Alternative potential modalities that were part of the intervention were: support groups, vouchers, phone or door-to-door contact by CHWs. Those external to the intervention were: newspaper, television, radio, *Baraazas* (community gatherings) or religious services. With regards to equity in access across interventions, we hypothesized that marginalized groups (defined as women, elderly, and those with lower income) are more likely to access door-to-door CHWs, religious services, or *Baraazas* than alternative modalities of contact: attending support groups, vouchers, television, radio, newspaper, or receiving SMS. It was postulated that the former modes are readily available to the marginalized individual, whereas the latter modes of contact require greater decision-making capacity or resources to access them. Some barriers include lack of time (children, housekeeping responsibilities), lack of empowerment, and financial restraints [12, 28, 29].

### Descriptive analysis

The characteristics of respondents (*Population Baseline* survey sample) were described using frequency, mean, median and percentage proportions. The number of respondents that answered individual questions in the secondary analysis varied, and is presented by result.

### Univariate analysis

Univariate analyses were done first to assess the association between socioeconomic, differential vulnerability, and differential exposure determinants [11, 12], and the two outcomes: willingness to receive, and whether or not SMS was received (reported in the *Clinic Baseline* and *Endline* surveys). The pre-determined factors in **Fig 2**: age, sex, level of education, level of income, “main source of livelihood” (type of work), number of hours worked and “quality of air in home” (home environment) were assessed using logistic regression analyses for association with the two outcome variables; Wald test statistic (p values) are presented in tables, and likelihood ratio test statistics (p values) for evidence of association are reported in the text. Where ordered categorical variables were present in the univariate analysis with apparent observations of trends in association with our outcomes of interest, these observations were further analyzed with a test for trend, using the likelihood ratio test.

Finally, for comparing equity across different modalities of health information, the odds for accessing SMS versus alternative modalities was compared across the independent variables: age, sex and level of income.

### Multivariable analysis

Independent variables, age and level of income, which were found to have a statistically significant association with willingness to receive SMS were included in the final model to adjust for potential confounding. Conversely, all of the sociodemographic factors, except for “quality of air in home” (indicator of social environment), were found to be associated with our second outcome, whether or not SMS was actually received, in univariate analysis. These were then considered as mentioned for the first outcome in a final model incorporating all associated variables. After the two multivariable models (for willingness to receive SMS and whether or not SMS was received) were determined, including independent variables that were statistically associated with the outcomes also being considered as potential confounders, the individual independent variables included in the models were tested for any evidence of interaction on the outcomes using the likelihood ratio test.

## Results

A total of 7,618 individual participants responded to one or more of the questions of interest for this secondary analysis from the four surveys. Participant ages ranged from 34 years old to 104 years old, with a median age of 44 years, and an average age of 47.2 years given a few significantly older outliers in the sample. The predominant age bracket of respondents was between 40 and 49 years old (40%, n = 2,268/ 5,635) as shown in **Table 1**. There was a minority of female respondents (n = 2,576), making up 45% of the sample.

**Table 1:** Characteristics of study population.

	Number of respondents in category (n)	Proportion of respondents (%)
**Age (years) of respondents**	** **	
Younger than 40	1,531	27.17
40–49	2,268	40.25
50–59	1,121	19.89
60–69	469	8.32
70–79	163	2.89
Older than 80	83	1.47
***Total***	***5*,*635***	***100***
		
**Sex of respondents**	** **	
Female	2,576	45.21
Male	3,122	54.79
***Total***	***5*,*698***	***100***
		
**Ever been to school**	** **	
Yes	4, 926	86.66
No	758	13.34
***Total***	***5*,*684***	***100***
		
**Level of education (grade) of respondents**	** **	
Less than primary school	983	19.95
Primary school	2,708	54.96
Secondary/ High School	1,139	23.12
College/ Pre-University/ University	96	1.95
Post graduate degree	1	0.02
***Total***	***4*,*927***	***100***
		
**Level of income (Kshs) of respondents**	** **	
Less than 1,000	315	6.65
1,000 to 2,499	776	16.39
2,500 to 4,999	1,254	26.48
5,000 to 7,499	1,115	23.54
7,500 or greater	1,276	26.94
***Total***	***4*,*736***	***100***
		
**Type of work**		
Unestablished own business (Informal)	2,062	36.28
Established own business (formal)	131	2.3
Informal salaried	487	8.57
Formal salaried	372	6.54
Informal casual	1,511	26.58
Formal casual	258	4.54
Rural agriculture	13	0.23
Urban agriculture	37	0.65
None currently	740	13.02
Other	73	1.28
***Total***	***5*,*684***	***100***
		
**Number of hours worked**		
Less than 8 hours	1,085	26.42
8–10 hours	2,340	56.98
Greater than 10 hours	682	16.61
***Total***	***4*,*107***	***100***
		
**Quality of air in home**		
Very High	282	4.98
High	1,349	23.8
Moderate	2,617	46.18
Low	1,024	18.07
Very low	395	6.97
***Total***	***5*,*667***	***100***

The majority of respondents reported attending school (87%, n = 4,926/ 5,684), however 75% (n = 3,691/ 4,927), had only completed up to primary (elementary) school. The majority of respondents reported earnings in the “KShs 7,500 or greater” income bracket (27%, n = 1,276/ 4,736). The most common livelihood was “Unestablished own business” (or “Informal”) constituting over one third of the population (36%, n = 2,062/ 5,684). These are individuals that run their own business but are not formally registered and not paying taxes. Only 2% of the population (n = 131/5,684) owned their own business (“formal”). Nearly one in five (17%, n = 682/4,107) reported working greater than ten hours per day.

### Equity factors affecting willingness to receive SMS

There was near universal willingness to receive SMS (94%, n = 568/604) among respondents (*Clinic Baseline*) Survey. Those willing to receive SMS were predominantly between 40–80 years old, to have at least a primary school education, to earn higher wages (greater than KShs 2,500), and to be economically active (working more than 8 hours per day).

When assessing the relationship between age and willingness to receive SMS, we found weak evidence for an association (p = 0.05), with age measured on six levels. The age groups most willing to receive SMS in the study were those aged between 40 and 49 years old (97%, n = 127/131), and 50–59 years old (96%, n = 126/131), respectively.

There was weak evidence of an association between level of income and willingness to receive SMS with level of income on five levels shown in **Table 2** (p = 0.04). Those with a lower income were less willing to receive SMS. There was very strong evidence of a linear trend (likelihood ratio test for trend, p = 0.009), with likelihood of being willing to receive SMS nearly doubling with each increase in income bracket (OR = 1.78, p = 0.01, 95% CI 1.12–2.81).

**Table 2:** Univariate analysis assessing association between equity factors and "Willingness to receive SMS".

	Number of respondents in category (n)	Number of respondents willing to receive SMS (%)	Odds Ratio: Willingness to receive SMS (95% CI, two-tailed)*	*P*-value**
**Age (years) of respondents**	** **			
Younger than 40	31	28 (90.32%)	1.00 *(reference group)*	
40–49	131	127 (96.95%)	3.40 (0.72–16.06)	0.12
50–59	131	126 (96.18%)	2.70 (0.61–11.97)	0.20
60–69	88	83 (94.32%)	1.78 (0.40–7.92)	0.45
70–79	48	45 (95.74%)	2.41 (0.38–15.34)	0.35
Older than 80	22	17 (77.27%)	0.36 (0.08–1.72)	0.20
				
**Sex of respondents**	** **			
Female	262	245 (93.51%)	1.00 *(reference group)*	
Male	188	181 (96.28%)	1.80 (0.73–4.42)	0.20
				
**Level of education (grade) of respondents**	** **			
Less than primary school	103	96 (93.20%)	1.00 *(reference group)*	
Primary school	175	168 (96.00%)	1.75 (0.60–5.14)	0.31
Secondary School	54	53 (98.15%)	3.86 (0.46–32.26)	0.21
				
**Level of income (Kshs) of respondents**	** **			
Less than 1,000	38	35 (92.11%)	1.00 *(reference group)*	
1,000 to 2,499	80	71 (88.75%)	0.68 (0.17–2.70)	0.58
2,500 to 4,999	109	106 (97.25%)	3.03 (0.59–15.70)	0.19
5,000 to 7,499	66	65 (98.48%)	5.57 (0.56–55.6)	0.14
7,500 or greater	47	46 (97.87%)	3.94 (0.39–39.54)	0.24
				
**Type of work**				
Own business (formal or informal)	217	204 (94.01%)	1.00 *(reference group)*	
Salaried	48	47 (97.92%)	3.00 (0.38–23.46)	0.30
Casual	78	76 (97.44%)	2.42 (0.53–10.98)	0.25
None	98	91 (92.86%)	0.83 (0.32–2.15)	0.70
				
**Number of hours worked**				
Less than 8 hours	131	123 (93.89%)	1.00 *(reference group)*	
8–10 hours	132	128 (96.97%)	2.08 (0.61–7.09)	0.24
Greater than 10 hours	53	52 (98.11%)	3.38 (0.41–27.73)	0.26
				
**Quality of air in home**				
Very High	16	14 (87.50%)	1.00 *(reference group)*	
High	82	78 (95.12%)	2.79 (0.47–16.69)	0.26
Moderate	225	217 (96.44%)	3.88 (0.75–20.00)	0.11
Low	80	73 (91.25%)	1.49 (0.28–7.93)	0.64
Very low	45	42 (93.33%)	2.00 (0.30–13.22)	0.47

*Univariate Odds Ratios comparing the odds of being "Willing to receive SMS" for each non-baseline group to the odds in the baseline ("reference") group

**P values represent results of Wald test for the Odds Ratios comparing individual levels to baseline ("reference") group within each variable

### Final models assessing willingness to receive SMS controlling for confounding and effect modification

After univariate analysis, only age and level of income were found to be statistically associated with the outcome, “willingness to receive SMS” (**Table 3**). After controlling for level of income, there was even stronger evidence of an association between age and willingness to receive SMS (p = 0.03). And after controlling for age, there was still similar weak evidence of an association between level of income and willingness to receive SMS (p = 0.04). This supports level of income as being a weak determinant on willingness to receive SMS in this sample. Lastly, in assessing for interaction between age and level of income, on willingness to receive SMS, there was no statistically significant evidence of interaction (p = 0.40).

**Table 3:** Multivariable regression analysis: Adjusted Odds Ratios after controlling for confounding of relationship between independent variables, "Age" and "Level of income", and outcome "Willingness to receive SMS".

	Adjusted OR Willingness to receive SMS (95% CI, two-tailed)*	*P*-value*
Age (years) of respondents**	-	-
Younger than 40	1.00 (*reference group*)	
40–49	3.71 (0.74–18.70)	0.11
50–59	2.37 (0.47–11.98)	0.30
60–69	3.71 (0.55–25.02)	0.18
70–79	0.91 (0.12–6.80)	0.93
Older than 80	0.15 (0.01–1.51)	0.11
				
Level of income***		
Less than 1,000	1.00 (*reference group*)	
1,000 to 2,499	0.58 (0.14–2.36)	0.44
2,500 to 4,999	2.70 (0.51–14.30)	0.24
5,000 to 7,499	5.00 (0.49–50.63)	0.17
7,500 or greater	3.45 (0.34–35.30)	0.30

*P values represent results of Wald test for the Odds Ratios comparing individual levels to baseline ("reference") group within each variable

**Odds Ratios for Age after controlling for level of income

***Odds Ratios for Level of Income after controlling for Age

### Equity factors affecting likelihood of SMS being received

In contrast to the majority of respondents demonstrating willingness, only 38% reported having received SMS (n = 192/ 507) in the *Clinic Endline* survey. Those that reported receiving SMS were more commonly in older age groups, 50 years and older, to be female, to have reported lower earnings (highest proportion amongst those earning less than KShs 1,000), and to work fewer hours (10 or less) or to be unemployed (**Table 4**).

**Table 4:** Univariate analysis assessing association between equity factors and "Whether or not received".

	Number of respondents in category (n)	Number of respondents who received SMS (%)	Odds Ratio: SMS was actually received (95% CI, two-tailed)*	*P*-value**
**Age (years) of respondents**	** **			
Younger than 40	23	20 (13.04%)	1.00 *(reference group)*	
40–49	120	36 (30.00%)	2.86 (0.80–10.22)	0.11
50–59	120	41 (34.17%)	3.46 (0.97–12.33)	0.06
60–69	75	31 (41.33%)	4.70 (1.28–17.19)	0.02
70–79	30	17 (56.67%)	8.72 (2.12–35.78)	0.01
Older than 80	16	13 (18.75%)	28.89 (5.04–165.58)	<0.001
				
**Sex of respondents**	** **			
Male	146	41 (28.08%)	1.00 *(reference group)*	
Female	238	100 (42.02%)	1.86 (1.19–2.89)	0.006
				
**Level of education (grade) of respondents**	** **			
Less than primary school	96	48 (50.00%)	1.00 *(reference group)*	
Primary school	151	44 (29.14%)	0.41 (0.24–0.70)	0.001
Secondary School	45	10 (22.22%)	0.29 (0.13–0.64)	0.002
				
**Level of income (Kshs) of respondents**	** **			
Less than 1,000	37	19 (51.35%)	1.00 *(reference group)*	
1,000 to 2,499	71	17 (23.94%)	0.30 (0.13–0.69)	0.005
2,500 to 4,999	97	33 (34.02%)	0.49 (0.23–1.05)	0.07
5,000 to 7,499	51	20 (39.22%)	0.61 (0.26–1.44)	0.26
7,500 or greater	36	7 (19.44%)	0.23 (0.08–0.65)	0.006
				
**Type of work**				
Own business (formal or informal)	191	63 (32.98%)	1.00 *(reference group)*	
Salaried	35	5 (14.29%)	0.34 (0.13–0.91)	0.03
Casual	73	29 (39.73%)	1.34 (0.77–2.34)	0.31
None	78	39 (50.00%)	2.03 (1.19–3.47)	0.01
				
**Number of hours worked**				
Less than 8 hours	109	36 (33.03%)	1.00 *(reference group)*	
8–10 hours	120	49 (40.83%)	1.40 (0.82–2.40)	0.22
Greater than 10 hours	45	8 (17.78%)	0.44 (0.19–1.04)	0.06
				
**Quality of air in home**				
Very High	13	4 (30.77%)	1.00 *(reference group)*	
High	72	25 (34.72%)	1.20 (0.33–4.28)	0.78
Moderate	178	59 (33.15%)	1.12 (0.33–3.77)	0.86
Low	75	34 (45.33%)	1.87 (0.53–6.59)	0.33
Very low	45	18 (40.00%)	1.50 (0.40–5.62)	0.55

*Univariate Odds Ratios comparing the odds of being "Willing to receive SMS" for each non-baseline group to the odds in the baseline (“reference”) group

**P values represent results of Wald test for the Odds Ratios comparing individual levels to baseline ("reference") group within each variable

There was very strong statistical evidence of an association between age and whether or not SMS was received (p<0.001) and to support a linear relationship with an increase in age (OR1.56, p<0.001, 95% CI 1.30–1.88). There was also very strong evidence of an association between sex and whether or not SMS was received with women having almost double the odds of receiving SMS as compared to males (OR 1.86, 95% CI 1.19–2.89, p = 0.006).

There was moderate statistical evidence of an association between level of income and whether or not SMS was received (p = 0.02). The odds increased with each increase in age bracket until the highest income bracket, KShs 7,500 or greater. There was very strong evidence of an association between level of education and whether or not SMS was received (p <0.001). Those with the least amount of education were most likely to have received the SMS (OR 0.49, p <0.001, 95% CI 0.34–0.72).

Additionally, we found very strong evidence (p = 0.009) of an association between type of work (measured on four levels, arrange in ordered fashion from most autonomous and stable, “self-owned business”, to least autonomous). Those that had no source of livelihood had twice the odds of receiving SMS as compared to those with their own business (OR 2.04, 95% CI 1.19–3.47, p = 0.01).

There was strong evidence of an association between number of hours worked and whether or not SMS was received (p = 0.02), with those working more than 10 hours having lower odds of receiving SMS compared to those working less than 8 hours (OR 4.44, 95% CI 0.19–1.04, p = 0.06). There was no evidence of an association with quality of air in home (proxy for social environment) and whether or not SMS was received (p = 0.43).

### Final models assessing whether or not SMS was received after controlling for confounding and effect modification

The final model assessing whether or not SMS was received included independent variables age, sex, level of income, level of education, type of work and number of hours worked (see **Table 5**). After controlling for confounding, estimates of the effects of sex, and number of hours worked, on willingness to receive SMS increased whereas the effect of age went down. Statistical evidence for association with level of income, level of education, and source of livelihood disappeared. Furthermore, there was no statistical evidence of effect modification of sex or level of income on the outcome. There was insufficient data to test for effect modification of age, education, type of work and number of hours worked.

**Table 5:** Multivariable regression analysis: Adjusted Odds Ratios after controlling for confounding of relationship between independent variables, "Age", Sex, "Level of education", "Level of income", "Type of work", and Number of Hours worked and outcome "Whether or not SMS was received".

	Adjusted OR Whether or not SMS was received (95% CI, two-tailed)	*P*-value*
**Age (years) of respondents**	-	-
Younger than 40	1.00 (*reference group*)	
40–49	2.44 (0.45–13.11)	0.30
50–59	3.23 (0.57–18.29)	0.19
60–69	9.72 (1.47–64.28)	0.02
70–79	13.87 (0.75–255.16)	0.08
Older than 80	ins**	
				
**Sex of respondents**		
Male	1.00 (*reference group*)	
Female	2.31 (1.03–5.21)	0.04
				
**Level of education (grade) of respondents**		
Less than primary school	1.00 (*reference group*)	
Primary school	0.66 (0.31–1.42)	0.29
Secondary School	0.40 (0.13–1.28)	0.12
				
**Level of income (Kshs) of respondents**		
Less than 1,000	1.00 (*reference group*)	
1,000 to 2,499	0.39 (0.12–1.32)	0.13
2,500 to 4,999	0.61 (0.20–1.89)	0.40
5,000 to 7,499	0.51 (0.14–1.85)	0.30
7,500 or greater	0.53 (0.11–2.48)	0.42
				
**Type of work**		
Own business (formal or informal)	1.00 (*reference group*)	
Salaried	0.30 (0.06–1.58)	0.15
Casual	1.76 (0.82–3.78)	0.15
None	ins**	
				
**Number of hours worked**		
Less than 8 hours	1.00 (*reference group*)	
8–10 hours	2.49 (1.17–5.33)	0.02
Greater than 10 hours	0.27 (0.06–1.15)	0.08

*P values represent results of Wald test for the Odds Ratios comparing individual levels to baseline ("reference") group within each variable

**ins: insufficient data in category to include in analysis (excluded from model by STATA due to insufficient observations in category)

### Equity in access to different aspects of the intervention and other health education modalities as compared to SMS

When comparing across our first marginalized population (the elderly), SMS was the health education modality with the highest odds of being accessed (OR 4.70, 8.72 and 28.89, for age brackets 60–69, 70–79 and 80 years or older, respectively). This was especially true in older age groups, 60 years or greater, where the odds of receiving SMS was five times higher or greater as compared to their younger counterparts. For other sources of health information, television (OR 5.07, 95% CI 2.15–11.93), and newspaper (OR 5.83, 95% CI 1.38-.24.59) had similar odds for the 60–69 year age group. This was in contradiction to our hypothesis that the elderly would be more likely to access more traditional forms of health information such as *Baraazas* or CHWs.

We hypothesized the same findings for the second considered marginalized population (women), but also found that they had the highest odds of accessing SMS over alternative aspects of the intervention (OR = 1.86, p = 0.006, 95% CI 1.19–2.89). However, there were somewhat similar odds of accessing door-to-door CHW meetings (OR 1.72, p<0.001, 95% CI 1.39–2.12). Of note, they had a lower odds of accessing *Baraazas* as compared to SMS, which was also in contradiction to our hypothesis. We did hypothesize less likelihood of being able to access support groups given these were outside the home and require autonomy to attend.

Outside of the intervention, newspapers (OR 3.26, 95% CI 2.11–5.04) were the most likely means of accessing health information for women. SMS access was similar as compared to radio and television (OR 1.86, 1.87, 1.65, respectively).

Finally, when assessing aspects of the intervention with greatest access by lower income respondents, these were: *Baraazas*, followed by SMS and lastly by door-to-door CHW meetings. Regarding other sources of health information, the odds of accessing SMS (OR 0.23, 95% CI 0.08–0.65) was similar to radio (OR 0.29, 95% CI 0.16–0.53), and television (OR 0.28, 95% CI 0.13–0.64). The newspaper was the source of health information with the highest odds for lower income respondents, whereas posters or flyers were least likely.

## Discussion

In our study, we found that the majority of participants, 94%, were willing to receive SMS, but only 38% reported actually receiving them. According to the WHO and Whitehead frameworks we used specific indicators to assess for equity of access to SMS [11, 12]: age, sex, level of education, level of income, work and home “exposures”. Within this framework, we were particularly interested in marginalized populations: those at the extremes of age, women, those with lower levels of education and income, and those with the worst working and living conditions. In line with our hypothesis, those working longer hours were less likely to receive SMS. In contradiction to our hypothesis, women, older respondents, those with lower income levels, and those with lower levels of education were more likely to receive SMS. Similarly, those with no work, and those working casual jobs, were more likely than their counterparts with self-owned businesses to have received the SMS. These findings demonstrate that SMS-based interventions can provide an equitably accessible modality for health interventions, and even potentially a preferred modality for marginalized populations with the poorest access to care.

With regards to assessing equity of access to SMS as compared to other healthcare information modalities, we found SMS was a strongly preferred modality among these marginalized populations, and was similarly accessed in comparison to newspaper and television. Women had similar odds of accessing SMS as compared to CHWs in the intervention, but the newspaper was the most common source for health information accessed. Women were least likely to access community health gatherings, support groups, and religious services. These additional findings may be indicative of urbanization, where such community gatherings are less likely to be attended [30]. Finally, we found that *Baraazas* were the most likely accessed aspect of the intervention by lower income groups, followed by SMS, and lastly by CHW interactions.

### Strengths and limitations

This is the first study to assess equity in mHealth use in the African setting, which provides critical information for its use and expansion in global public health. We look at marginalized populations, and identify the applicability of mHealth not only as compared to standard forms of media, but also other routinely used modalities in public health, particularly *Baraazas* and CHWs. This information is key in developing public health interventions as we seek to optimize strategies for increased effectiveness in our target populations.

As expected with a secondary analysis, there were limited responses in certain questions and missing values, which limited power in running some of our analyses. Where trends existed, we addressed this, and anticipate that further prospective studies would better delineate these findings.

Additionally, some of the questions appeared to be poor discriminators as indicators. The question on whether or not SMS was received does not differentiate whether or not the SMS was delivered, was delivered and went unread by the respondent, or if it was never transmitted at all. All the same, we posit the most important effect represented by the question is whether or not the SMS made an impression on the recipient. These same barriers (errors in transmission or messages going unaccessed despite delivery to device) in implementation of mHealth interventions would also be expected. Questions to be used as indicators of “work and social environment” were used that may not have directly answered the question, particularly, the question on “quality of air in environment”. More specific questions to address these sociodemographic factors would have been ideal.

There is also the concern of recall bias, particularly in the case of the Endline surveys. Additionally, responder bias may have occurred, given the surveys were conducted as face-to-face interviews. This may have been especially in the case of particularly sensitive questions, like level of education, level of income (257 respondents reported “I don’t know”), and type of work. We, however do not feel that this affected our results significantly, as secondary data from demographic surveillance surveys demonstrate similar population distribution [31].

Potential confounding affecting willingness to receive SMS that are not accounted for in the study include the respondent’s level of comfort with SMS, level of trust for the program organization, providers or study team, fear of poor quality of care and lack of engagement associated with poorer populations overall [19, 32–34]. Additionally, those with lower SES tend to have worse health indicators and are more susceptible to chronic disease. Given care was provided as part of the SCALE UP intervention, this may have caused study or responder bias given perceived benefits of receiving healthcare based on survey responses [12].

Finally, given the sample is representative of a slum population, findings may only be generalizable to slums in similar urban settings. Our sample population was felt to be generalizable to the general slum with similar demographic characteristics, as shown in the Nairobi Urban Health Demographic Surveillance System (NUHDSS) [30, 31].

### Marginalized populations more likely to access SMS

Women though typically seen as lacking empowerment in the household were, contrary to our hypothesis, more likely to access SMS than men in our study. One reason that this might have occurred is that women, having greater concern with healthcare, may engage more with these health-related texts [35]. An alternative consideration is that given the African household is more likely to own only one mobile phone, the wife may be more likely to stay at home with it and therefore be more likely to access messages [36]. Given its use by women in our study, targeting maternal and child health issues through mHealth may be opportune.

We were also surprised by findings in the elderly population, given concerns for lack of understanding or fear of use of technology. However, the elderly may have been more available to access text messages given they are less distracted by other activities, such as work and childcare. They also may already have chronic disease, and thus be more motivated to access texts and care in the intervention.

Contrary to hypothesis, the youngest age groups (in addition to the oldest ones) were least willing to receive SMS. While it was encouraging to find that those in the age groups forty to sixty years old, who are affected by NCDs, were most willing to receive SMS in our study, attention needs to be paid to the youngest group (those under 40 years old) given prevention at this age is critical and cost-effective [37, 38, 39]. Attention should be given with the introduction of mHealth to provide education, awareness and support to these groups. Increasing patient involvement in care through mHealth has been shown to improve health outcomes. This is done through: goal setting and collaborative planning with the care team, allowing the patient to interact with and track achievements, and distribution of important lifestyle changes or risk reduction achievements as they happen [18, 40].

While those with lower levels of income reported being less willing to receive SMS, which was in line with our hypothesis, they actually had a higher odds of receiving SMS as compared to wealthier counterparts. Some barriers to willingness of use of mHealth in this group might be perceived resources (time or money) involved in accessing SMS [25, 41]. In approaching these groups, sensitization should be provided to improve uptake of mHealth interventions. Finally, lack of education was not a barrier to accessing mHealth; those with higher levels of education were less likely to have received SMS. This shows promise for SMS in engaging even those with less education in health interventions using technology.

### Implications for mHealth implementation research and policy development

Timely and appropriate response to the problem of NCDs in the developing world is needed, and the use of mobile health technology is proposed as an innovative means of contributing to the solution. The use of mHealth is a key component in increasing participation of the patient in chronic management of NCDs, a concept that has been shown to improve outcomes in chronic disease patients in high income countries [13, 14, 42–48].

Overall, the potential reach of mHealth interventions on women is paramount, and studies or policy targeting this group should be prioritized moving forward. Our findings showing that those working more than 10 hours, representative of men, further credits women as a target for mHealth interventions. This also suggests that critical health interventions targeting men may warrant alternative approaches including workplace interventions.

Further studies are needed to better understand limitations for access to mHealth, including further exploration of the disparity between those willing to receive SMS (94%) versus those that actually received the SMS (38%) demonstrated in our study. These analyses should be done in larger sample populations. Additionally, further studies on equity in mHealth in urban populations outside of slums, and in rural populations where some of the needs and access issues are greatest, as well as disease-specific effects of mHealth are needed [12, 39].

Particular innovation is needed for slums given that populations are exponentially increasing in size, including in Nairobi, Kenya, the location of our study, where the slum population has risen from 350,000 in 1962 to almost 3.5 million in 2009 [30]. In the SCALE UP intervention, employment of mHealth was through the use of text messages which has been shown to allow for greater “anonymity” and potential for “personalization”, which may have been reflected in a remarkable 94% response rate of willingness to receive SMS [44, 49, 50].

Finding the youngest population in our sample to be less likely to access mHealth in our intervention supports the notion that findings in developed nations are not necessarily generalizable to LMIC settings. These findings contradict existing literature from developed nations, which demonstrates those that are younger are more likely to use mobile technologies [51]. This emphasizes the need for studies in the LMIC setting.

## Conclusion

In summary, women and those working fewer hours have the greatest likelihood of receiving SMS in our intervention. The elderly, women and lower income populations, were more likely to access SMS as compared to alternative modalities of health information such as CHWs, newspaper, television and radio. Mobile phones do not have the capacity to deliver many of the health care interventions currently provided by CHW but our results suggest they may have higher reach in disseminating information and education. Sensitization prior to implementation of mHealth interventions may need to be provided to elderly and lower income individuals. Furthermore, for those working long hours, alternative or additional modes outside of mHealth should be considered, for example through targeted work-place interventions. These research questions provide guidance for intervention development and further study in targeting mHealth towards marginalized populations in the African slum population and beyond.
